# Complete Genome Sequences of Five Salmonella enterica Phages, NBSal001, NBSal002, NBSal003, NBSal004, and NBSal005

**DOI:** 10.1128/MRA.00301-20

**Published:** 2020-05-28

**Authors:** Carlos D. Llanos, Jaime Ortega, Jorge A. Bardales

**Affiliations:** a Nextbiotics, Inc., Oakland, California, USA; Queens College

## Abstract

Salmonella enterica is a Gram-negative bacterium, recognized as one of the most important foodborne pathogens in the world. Bacteriophages represent a promising alternative to the biocontrol of *Salmonella*. Here, we report the isolation of five *Salmonella* bacteriophages, the sequencing of their full genomes, and initial genomic characterization.

## ANNOUNCEMENT

In this study, we report the complete genome sequences of five lytic phages, NBSal001, NBSal002, NBSal003, NBSal004, and NBSal005. The lytic phages were isolated from poultry waste samples by using different Salmonella enterica serovars with the soft-agar overlay method (1). Bacterial and phage cultures were isolated and amplified by incubating the samples for 18 h at 37°C in tryptic soy broth or agar. The phage genomes were purified using a modified phenol-chloroform method to enhance their purity (2). The phage genomes were prepared for sequencing by using a QIAseq FX single-cell DNA library kit and sequenced using the MiSeq Illumina platform. The raw reads were trimmed using Trimmomatic (3), resulting in more than 3 million paired-end reads with a length of 151 nucleotides for each genome assembly (Table 1). With a previous quality evaluation with FastQC, the assembly was performed using the SPAdes genome assembler v3.9.0 (4). The phage genome ends were determined using the PhageTerm tool (5). Then, genome annotation was performed using the RAST server, and the annotation was manually curated with the NCBI protein database (6). Finally, the tRNAs were identified using tRNAscan-SE v2.0, included in the RAST server (7). Default parameters were used for all software unless otherwise specified. The phage genomes showed coverages above 1,000× and lengths ranging from 50,922 bp to 121,040 bp, with an average GC content of 39.9%. The packaging strategies identified in these phages were direct terminal repeats (short and long) for nonpermutated genomes but were not identified for permutated genomes. The number of genes with predicted functions varies from phage to phage, as does the presence of tRNAs. The proximity to other phage genomes, evaluated through nucleotide BLAST, showed notable similarity with previously described phages; in particular, most of the phages were related to the *Siphoviridae* family, with the exception of phage NBSal004, which is related to the *Myoviridae* family. These characteristics are more precisely explained in Table 1.

**TABLE 1:** Characteristics of phages NBSal001, NBSal002, NBSal003, NBSal004, and NBSal005

Phage name (accession no.)	No. of paired-end reads (million)	Genome length (bp)	Coverage (×)	Packaging strategy	No. of genes	No. of genes with predicted functions	No. of tRNAs	GC content (%)	Closest phage (accession no., % identity)
Phage NBSal001 (MN994500)	3.1	50,922	2,096	Unknown	78	56	0	42.2	Phage FSL SP-126 (NC_042066, 92.16)
Phage NBSal002 (MN994501)	3.5	120,250	1,125	DTR^*a*^ (long)	178	151	21	40.1	Phage BSP22A (KY787212, 99.73)
Phage NBSal003 (MN994502)	3.4	119,416	1,107	DTR (long)	170	150	18	39.6	Phage S131 (MH370378, 95.34)
Phage NBSal004 (MN994503)	3.9	86,841	1,546	DTR (short)	130	124	20	38.8	Phage VSe11 (MG251391, 95.94)
Phage NBSal005 (MN994504)	3.5	121,040	1,092	DTR (long)	178	156	19	39.1	Phage vB_EcoS_FFH_1 (NC_024139, 96.12)

a DTR, direct terminal repeat.

The exploration of biocontrol alternatives for foodborne pathogens such as Salmonella enterica is of the utmost importance for human health and wellness. Here, we present the genomes of five highly lytic bacteriophages with the potential to be used in the biocontrol of multiple Salmonella enterica serovars.

### Data availability.

The genome sequences and raw data have been deposited in GenBank under the accession numbers MN994500 and SRR11341260 (NBSal001), MN994501 and SRR11341346 (NBSal002), MN994502 and SRR11341398 (NBSal003), MN994503 and SRR11341420 (NBSal004), and MN994504 and SRR11341467 (NBSal005), respectively.

## References

[1] Adams MH 1956 Bacteriophages. Interscience Publishers, Inc, New York, NY.

[2] Pickard DJJ 2009 Preparation of bacteriophage lysates and pure DNA, p 3–10. *In* Clokie MRJ, Kropinski AM (ed), Bacteriophages: methods and protocols, vol 2 Humana Press, New York, NY. 10.1007/978-1-60327-565-1_1 19082547

[3] Bolger AM, Lohse M, Usadel B 2014 Trimmomatic: a flexible trimmer for Illumina sequence data. Bioinformatics 30:2114–2120. doi:10.1093/bioinformatics/btu170. 24695404 PMC4103590

[4] Bankevich A, Nurk S, Antipov D, Gurevich AA, Dvorkin M, Kulikov AS, Lesin VM, Nikolenko SI, Pham S, Prjibelski AD, Pyshkin AV, Sirotkin AV, Vyahhi N, Tesler G, Alekseyev MA, Pevzner PA 2012 SPAdes: a new genome assembly algorithm and its applications to single-cell sequencing. J Comput Biol 19:455–477. doi:10.1089/cmb.2012.0021. 22506599 PMC3342519

[5] Garneau JR, Depardieu F, Fortier L-C, Bikard D, Monot M 2017 PhageTerm: a tool for fast and accurate determination of phage termini and packaging mechanism using next-generation sequencing data. Sci Rep 7:8292. doi:10.1038/s41598-017-07910-5. 28811656 PMC5557969

[6] Aziz RK, Bartels D, Best AA, DeJongh M, Disz T, Edwards RA, Formsma K, Gerdes S, Glass EM, Kubal M, Meyer F, Olsen GJ, Olson R, Osterman AL, Overbeek RA, McNeil LK, Paarmann D, Paczian T, Parrello B, Pusch GD, Reich C, Stevens R, Vassieva O, Vonstein V, Wilke A, Zagnitko O 2008 The RAST server: Rapid Annotations using Subsystems Technology. BMC Genomics 9:75. doi:10.1186/1471-2164-9-75. 18261238 PMC2265698

[7] Lowe TM, Chan PP 2016 tRNAscan-SE On-line: integrating search and context for analysis of transfer RNA genes. Nucleic Acids Res 44:W54–W57. doi:10.1093/nar/gkw413. 27174935 PMC4987944

